# Reversal Treatment in Oral Anticoagulant-Related Intracerebral Hemorrhage—An Observational Study Based on the Swedish Stroke Register

**DOI:** 10.3389/fneur.2020.00760

**Published:** 2020-07-29

**Authors:** Trine Apostolaki-Hansson, Teresa Ullberg, Mats Pihlsgård, Bo Norrving, Jesper Petersson

**Affiliations:** ^1^“Stroke Policy and Quality Register Research” Group, Department of Neurology, Skåne University Hospital, Lund University, Lund, Sweden; ^2^Department of Geriatrics, Skåne University Hospital, Lund University, Malmö, Sweden

**Keywords:** patient outcome, acute stroke, intracranial hemorrhage, mortality, survival, anticoagulants, prognosis, hemostatic techniques

## Abstract

**Introduction:** Intracerebral hemorrhage (ICH) is the most serious adverse effect of oral anticoagulant (OAC) treatment. The effect of OAC reversal therapy on outcome is uncertain. We compared 90-day survival and functional outcome in patients with OAC-ICH who received OAC reversal therapy with those who did not.

**Methods:** Data from The Swedish Stroke Register (Riksstroke) for all registered cases of OAC-ICH during 2017 (572 patients) were used to obtain information on reversal (*n* = 369) and non-reversal (*n* = 203) treatment receiving patients. Univariate and multivariate Cox regression analysis stratified for level of consciousness (LOC) on admission, and adjustment for relevant baseline variables, was used to compare 90-day Hazard Ratios (HR) for mortality.

**Results:** Sixty-five percent of patients received reversal treatment. These patients were younger, more often pre-stroke independent and alert at presentation. Withholding reversal treatment was associated with an increased death rate (*HR* = 1.47; 95% CI: 1.08–2.01) in a Cox regression model stratified for LOC and adjusted for baseline imbalances. Additional factors associated with an increased 90-day death rate were male sex (*HR* = 1.42; 95% CI: 1.06–1.92), age (*HR* = 1.05; 95% CI: 1.02–1.07), and intraventricular hemorrhage (*HR* = 2.41; CI: 1.77–3.29).

**Conclusion:** In this large observational study 35% of patients with OAC-ICH did not receive reversal treatment. Patients receiving OAC-reversal treatment had an improved 90-day mortality outcome compared to those not receiving treatment. Mortality was strongly related to LOC. Further, and larger, studies are required to determine which patient groups may benefit from reversal therapy and in whom non-reversal is adequate.

## Introduction

Intracerebral hemorrhage (ICH) is the most serious complication of oral anticoagulant therapy (OAC) with an approximate yearly incidence of 0.1–0.6% (1, 2). The increased use of OAC for the prevention of ischemic stroke in patients with non-valvular atrial fibrillation has led to an increased number of OAC-related ICH (3, 4). Prognosis after OAC-ICH is poor, with reported mortality rates reaching 45%, and functional dependency rates of 75% by 3 months, regardless of OAC type (5–8). However, recent data show that 30-day mortality rates following OAC-ICH may be lower in patients with non-vitamin K oral anticoagulant (NOAC)—related ICH compared to vitamin K antagonist (VKA)—related ICH (9). Factors predicting poor outcome and high early case fatality in OAC-ICH include hematoma expansion, large hematoma volumes, and intraventricular hemorrhage (10–14). Early administration of hemostatic therapy can inhibit hematoma expansion, yet the benefit of this measure on patient outcome is uncertain (15, 16).

The 2014 European Stroke Organization (ESO) guidelines on management of spontaneous intracerebral hemorrhage provided no recommendation regarding hemostatic treatment following OAC-ICH (17). However, reversal therapy for OAC-ICH is recommended in the ESO guidelines specifically on this topic published 2019 (18), although these recommendations are based on weak quality evidence. Vitamin K and four-factor prothrombin complex concentrate (PCC) are recommended for reversal of VKA activity (19, 20). Direct antidotes are recommended for the reversal of NOAC, and PCC is recommended in the absence of a specific antidote (20, 21).

Our study aims to delineate mortality and functional outcome at 90 days post-OAC-ICH by comparing individuals receiving OAC-reversal therapy to individuals not receiving reversal therapy in a large nationwide stroke cohort representing recent clinical practice in Sweden.

## Methods

### Database and Study Population

All patients >18 years who presented with ICH (ICD.10 I61.9) during ongoing anticoagulant treatment, and who were registered in The Swedish Stroke Register (Riksstroke) between January 1, 2017 and December 31, 2017 were included. Patients were anticoagulated prior to ICH with either VKA, Apixaban, Dabigatran, or Rivaroxaban.

Riksstroke is a nationwide hospital-based stroke quality register, with a coverage of >90% of hospital admitted stroke cases in Sweden (22). Patients were registered in Riksstroke during their hospital stay. Three-month follow-up data were attained from Riksstroke's follow-up questionnaire, which was distributed by mail, with two postal reminders to non-respondents. The follow-up questionnaire was either completed by the patient alone, with caregiver assistance, or by caregiver alone. Data on indications for anticoagulation therapy other than atrial fibrillation, reasons for withstanding reversal treatment, and laboratory values regarding NOAC serum concentrations were not available. All-cause mortality status was obtained from the Swedish Causes of Death Register, with a coverage of >98% (23). Patients who died within the 90 days were considered followed up.

### Outcome Variables

Baseline characteristics included: age, sex, vascular risk factors, history of previous stroke, or transitory ischemic attack (TIA), and pre-stroke dependency. Stroke care characteristics included: time interval between symptom onset to hospital arrival, hemorrhage location (supra- or infratentorial), presence of intraventricular hemorrhage, international normalized ratio (INR), neurosurgery, hemostatic drug, stroke unit/ICU admission, and median days of hospital admittance. Level of consciousness (LOC) at admission based on the reaction level scale (RLS-85) was used as a proxy for stroke severity. The RLS-85 is an eighth grade single line scale commonly used in Sweden to assess LOC, and correlates with the Glasgow Coma Scale (24). Riksstroke presents RLS-85 as three main categories: alert, drowsy, and comatose. Primary outcome variables were mortality and functional outcome at 90 days post-OAC-ICH.

At 3 months post-OAC-ICH, we used a modified Rankin Scale (mRS) score (translated using a previously validated translation algorithm) to assess patient-reported outcome variables on dressing and toileting (independent or assistance required), living conditions (living independently, living independently with homecare, residing at an assisted living facility or in need of in-patient care), mobility (fully mobile both indoors and outdoors, mobile but only indoors or fully dependent on assistance for mobility), and dependency on next of kin for support (fully dependent on relatives, partially dependent, not dependent) (25). The final mRS score was categorized as mRS 0–2 (independent), mRS 3–5 (dependent), or mRS 6 (deceased).

### Statistical Methods

Statistical analysis was performed using IBM SPSS Statistics version 24. Patient baseline characteristics were grouped according to those receiving OAC-reversal treatment vs. non-OAC reversed patients and are presented as simple proportions, medians and means. Independent samples *t*-tests and Mann-Whitney tests were used to analyze continuous variables. A Chi-squared test determined differences between categorical variables. Survival curves were constructed using the Kaplan-Meier method. In order to determine whether death rates were affected by OAC-reversal treatment, we conducted two Cox regression analyses; one simple and one adjusted for age, sex, diabetes, hypertension, atrial fibrillation, pre-stroke dependency, anticoagulant reversal, hemorrhage location (supratentorial vs. infratentorial), intraventricular hemorrhage, and neurosurgery. Due to the indication of non-proportionality in mortality rates corresponding to different LOC categories, a stratified model was applied to the multivariate analysis using LOC category as the stratification variable.

Simple and stratified multivariable linear regressions were also performed comparing mortality with and without reversal treatment for patients with VKA-ICH and NOAC-ICH, separately. Presenting INR level (<1.7, 1.7–3, >3) was included as a covariate in VKA-ICH multivariate analysis. A *p* <0.05 was considered statistically significant.

### Ethical Considerations

This study was approved by the local Research Ethics Committee in Lund, Sweden (dnr 2017/529). Anonymized data were used. Individual consent was not required for this study as patients were informed of the handling of their data for possible future research purposes on entry into the quality register. This article aligns with STROBE criteria for observational studies (26).

## Results

The study included 572 patients presenting with acute OAC-ICH during 2017. Of these, 369 patients were receiving OAC-reversal treatment (118 NOAC, 251 VKA) and 203 patients were not receiving OAC-reversal treatment (117 NOAC, 86 VKA).

### Patient Characteristics

Baseline patient characteristics and missing data are displayed in Table 1. Patients receiving reversal treatment were younger, more often pre-stroke independent and more likely treated with VKA than NOAC. Level of consciousness at hospital admission differed between groups: 65.0% presented as alert, 26.0% drowsy, and 9.0% comatose in the reversal treatment group, compared to 45.5, 23.8, and 30.7%, respectively, in the non-reversal treatment group (*p* <0.001). Time from symptom onset to hospital admission was similar in patients receiving reversal vs. non-reversal treatment, with most patients arriving within 3 h of symptom onset (*p* = 0.49). Irrespective of treatment group, drowsy and comatose patients were more frequently admitted within 3 h of symptom onset compared to alert individuals (*p* = 0.02). Patients receiving reversal treatment were more likely cared for in a stroke care unit or ICU setting (*p* = 0.001). Patient characteristics of those lost to follow-up are displayed in Supplemental Table 1.

**Table 1:** Baseline characteristics of 572 patients with OAC-ICH comparing patients that received reversal treatment compared to patients who did not receive reversal treatment.

**Variables**	**Reversal**	**Non-reversal**	***p*-value**
	**(*n* = 369)**	**(*n* = 203)**	
	***n* (%)**	***n* (%)**	
**Demographics**			
Mean age	79.0 (±9.2)^*^	81.4 (±8.9)^*^	0.003
Sex (male)	205 (55.6)	104 (51.2)	0.32
Pre-stroke dependent	116 (32.7)	94 (46.3)	<0.001
**Vascular risk factors**			
Hypertension	296 (80.2)	167 (82.3)	0.79
Atrial fibrillation	319 (86.4)	182 (89.7)	0.18
Diabetes	80 (21.7)	36 (17.8)	0.27
Previous stroke	98 (26.6)	73 (36.0)	0.02
Previous TIA	42 (11.4)	21 (10.3)	0.84
**Clinical characteristics**			
Symptom onset to hospital arrival (time)			0.49
*0–3 h*	141 (38.2)	88 (43.3)	
*3–6 h*	110 (29.8)	50 (24.6)	
*>6 h*	88 (23.8)	46 (22.7)	
Admitted to stroke unit or ICU	364 (90.1)	164 (80.8)	0.001
Length of hospital stay (median days)	11	5	<0.001
Level of consciousness at hospital admission			<0.001
*Alert*	238 (65.0)	92 (45.5)	
*Drowsy*	95 (26.0)	48 (23.8)	
*Comatose*	33 (9.0)	62 (30.7)	
**Hemorrhage location**			
Supratentorial	311 (85.2)	183 (91.0)	0.05
*Intraventricular hemorrhage*	*140/311 (45.8)*	*85/183 (47.5)*	0.71
*Neurosurgery*	*9/311 (2.9)*	*2/183 (1.1)*	0.19
Infratentorial	54 (14.8)	18 (9.0)	0.05
*Intraventricular hemorrhage*	*14/54 (26.9)*	*5/18 (27.8)*	0.94
*Neurosurgery*	*7/54 (13.0)*	*0/18 (0.0)*	0.11
**Anticoagulant**			
NOAC	118 (32.0)	117 (57.6)	
*Apixaban*	*78 (66.1)*	*83 (70.9)*	0.64
*Rivaroxaban*	*31 (26.3)*	*28 (23.9)*	
*Dabigatran*	*9 (7.6)*	*6 (5.1)*	
VKA	251 (68.0)	86 (42.4)	
*INR <1.7*	*16 (6.4)*	*10 (11.6)*	0.17
*INR 1.7–3*	*149 (59.4)*	*43 (50.0)*	
*INR >3*	*86 (34.3)*	*33 (38.4)*	

* *Standard deviation of the mean. OAC, oral anticoagulant; ICH, intracerebral hemorrhage; VKA, Vitamin K antagonist; NOAC, non-vitamin K oral anticoagulant; INR, international normalized ratio; ICU, intensive care unit; TIA, transitory ischemic attack*.

*Proportion of missing data varied between 0 and 1.0% for all variables except for VKA reversal type (2.3%), intraventricular hemorrhage (2.5%), pre-stroke dependency (4.5%), NOAC reversal type (11%), and time interval between symptom onset to hospital arrival (8.6%). The characteristics seen in italics comparing Supra vs infratentorial under hemorrhage location*.

### Reversal Treatment

In patients exhibiting VKA-ICH, 85.7% of reversal treatment cases were treated with PCC and Vitamin K, while 11.6% received PCC alone. In NOAC-ICH patients receiving reversal treatment, 82.2% were treated with PCC, 5.9% received Idarucizumab, and one patient received both PCC and Idarucizumab. Data on type of hemostatic agent were missing in 2.3% of VKA-ICH and 11% of NOAC-ICH cases.

### Cumulative Mortality

All 572 patients were included in the mortality analysis. All-cause mortality in patients receiving reversal treatment was 19.2% at 7 days, 30.1% at 30 days, and 33.6% at 90 days. The corresponding numbers for non-reversal treatment patients were 41.9, 48.8, and 52.7%, respectively. Mortality differed significantly between patients receiving OAC-reversal and non-reversal treatment at all endpoints when the simple Cox analysis was applied. At 90 days, Hazard ratio (HR) for death using the simple analysis was 1.92 (95% CI: 1.48–2.49; Table 2).

**Table 2:** Cox regression analysis stratified for level of consciousness showing Hazard Ratios (HR) for 90-day mortality in 572 patients with OAC-ICH.

**Variable**	**HR**	**95% CI**		***P*-value**
		**Lower**	**Upper**	
**Crude model (non-stratified)**				
No OAC-reversal	1.92	1.48	2.49	<0.001
Adjusted model (stratified)^*^				
No OAC-reversal	1.47	1.08	2.01	0.02
Male sex	1.42	1.06	1.92	0.02
Age	1.05	1.02	1.07	<0.001
Diabetes	1.03	0.70	1.50	0.89
Hypertension	0.80	0.57	1.14	0.22
Atrial fibrillation	0.74	0.48	1.14	0.17
Pre-stroke dependency	1.02	0.74	1.40	0.92
Intraventricular hemorrhage	2.41	1.77	3.29	<0.001
Neurosurgery not performed	2.13	0.91	5.02	0.08
Infratentorial hemorrhage	1.47	0.96	2.24	0.08

*Simple analysis is displayed as a crude model. OAC, oral anticoagulant; ICH, intracerebral hemorrhage; CI, confidence interval*.

* *Stratified for level of consciousness (alert, drowsy, and comatose)*.

**Figure 1:**
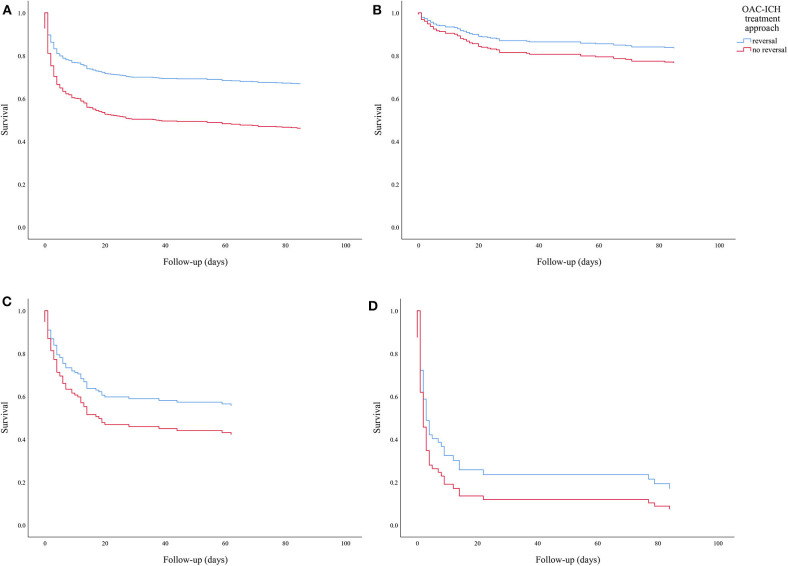
**(A)** Simple analysis showing Kaplan-Meier survival curve of 90-day cumulative survival following OAC-ICH comparing patients with reversal treatment vs. non-OAC reversal patients (*n* = 572). **(B–D)** Survival curves based on multivariate analysis stratified for level of consciousness, comparing 90-day cumulative survival following OAC-ICH in patients receiving reversal treatment vs. non-OAC reversed patients. State of consciousness according to graph is as follows: **(B)** alert (*n* = 316), **(C)** drowsy (*n* = 128), **(D)** comatose (*n* = 78).

All-cause mortality at 90 days differed according to presenting LOC, irrespective of reversal treatment, and was as follows: alert 18.4%, drowsy 53.1%, and comatose 89.0%. Figures 1A–D display the Kaplan-Meier survival curves. Multivariate Cox regression analysis stratified for LOC confirmed an increased risk of death in patients not receiving reversal treatment (*HR* = 1.47; 95% CI: 1.08–2.01; Table 2).

**Figure 2:**
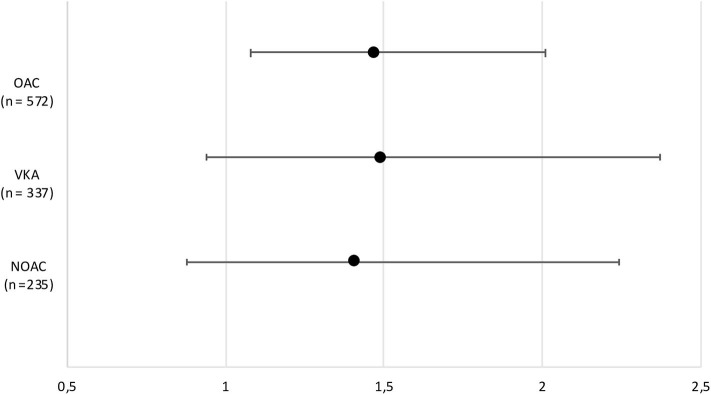
Forest plot demonstrating hazard ratios and confidence intervals for 90-day mortality in patients who did not receive reversal treatment following OAC related ICH, and in subgroup analyses regarding VKA-ICH and NOAC-ICH separately. ICH, intracerebral hemorrhage; VKA, vitamin K antagonist; NOAC, non-vitamin K antagonist oral anticoagulant.

In the VKA-ICH subgroup, HR for death in patients not receiving OAC-reversal treatment was 1.49 (95% CI: 0.94–2.37; Table 3A). Patients presenting with INR 1.7–3 had a death rate of 1.96 (95% CI: 0.69–5.54), and those presenting with INR values >3 had a death rate of 2.16 (95% CI: 0.75–6.25). In NOAC-ICH patients not receiving reversal treatment, HR for death was 1.41 (95% CI: 0.88–2.24; Table 3B and Figure 2). Ninety-day mortality did not differ between VKA vs. NOAC-ICH (NOAC *HR* = 0.95; 95% CI: 0.70–1.28; Supplemental Table 2).

**TABLE 3A:** Cox regression analysis stratified for level of consciousness showing Hazard Ratios (HR) for 90-day mortality in 337 patients with VKA-ICH.

**Variable**	**HR**	**95% CI**		***P*-value**
		**Lower**	**Upper**	
**Crude model (non-stratified)**				
No VKA reversal	2.20	1.54	3.13	<0.001
Adjusted model (stratified)^*^				
No VKA reversal	1.49	0.94	2.37	0.09
Male sex	1.42	0.93	2.16	0.10
Age	1.06	1.03	1.09	<0.001
Diabetes	1.68	1.01	2.79	0.05
Hypertension	0.68	0.44	1.06	0.09
Atrial fibrillation	0.53	0.28	1.01	0.05
Pre-stroke dependency	0.79	0.50	1.25	0.31
INR				
< *1.7*	1			
*1.7–3*	1.96	0.69	5.54	0.21
*>3*	2.16	0.75	6.25	0.15
Intraventricular hemorrhage	2.23	1.45	3.43	<0.001
Neurosurgery not performed	2.38	0.80	7.12	0.12
Infratentorial hemorrhage	1.37	0.78	2.39	0.28

*Simple analysis is displayed as a crude model. ICH, intracerebral hemorrhage; CI, confidence interval; VKA, vitamin K antagonist; INR, international normalized ratio*.

* *Stratified for level of consciousness (alert, drowsy, and comatose)*.

**TABLE 3B:** Cox regression analysis stratified for level of consciousness showing Hazard Ratios (HR) for 90-day mortality in 235 patients with NOAC-ICH.

**Variable**	**HR**	**95% CI**		***P*-value**
		**Lower**	**Upper**	
**Crude model (non-stratified)**				
No NOAC reversal	1.72	1.15	2.57	0.008
Adjusted model (stratified)^*^				
No NOAC reversal	1.41	0.88	2.24	0.15
Male sex	1.53	0.98	2.39	0.06
Age	1.03	1.00	1.07	0.04
Diabetes	0.57	0.32	1.05	0.07
Hypertension	1.17	0.65	2.13	0.60
Atrial fibrillation	0.94	0.49	1.79	0.84
Pre-stroke dependency	1.22	0.76	1.95	0.42
Intraventricular hemorrhage	2.82	1.75	4.53	<0.001
Neurosurgery not performed	1.63	0.38	7.03	0.52
Infratentorial hemorrhage	1.69	0.83	3.45	0.15

*Simple analysis is displayed as a crude model. ICH, intracerebral hemorrhage; CI, confidence interval; NOAC, non-vitamin K antagonist oral anticoagulant*.

* *Stratified for level of consciousness (alert, drowsy, and comatose)*.

### Functional Outcome

**Figure 3:**
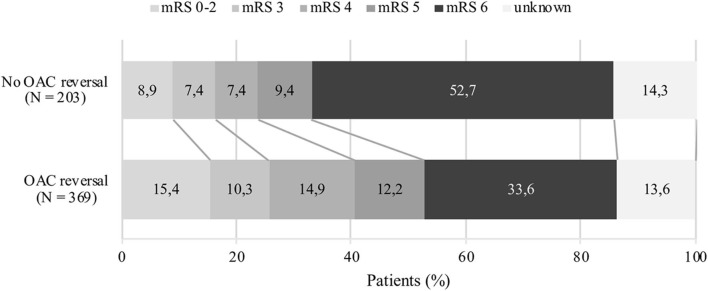
Crude data comparing 90-day functional outcome following oral anticoagulant related intracerebral hemorrhage. Figure includes patients lost to follow-up. VKA, vitamin K antagonist; NOAC, non-vitamin K antagonist oral anticoagulant.

**Figure 4:**
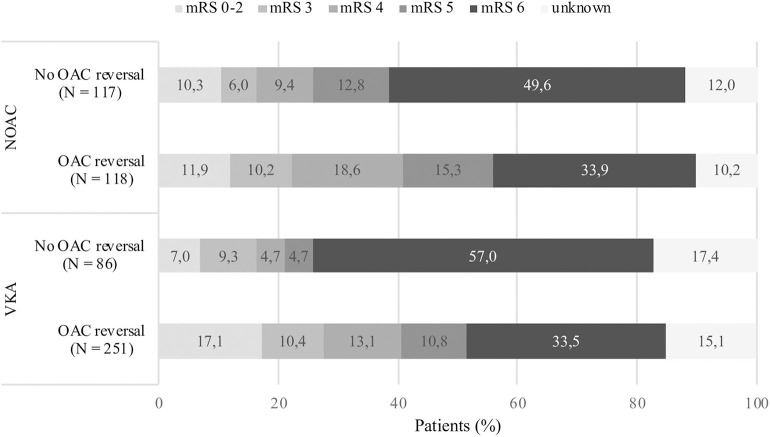
Crude data comparing 90-day functional outcome following oral anticoagulant related intracerebral hemorrhage. Figure includes patients lost to follow-up. ICH, intracerebral hemorrhage; VKA, vitamin K antagonist; NOAC, non-vitamin K antagonist oral anticoagulant.

Ninety-day functional outcome follow-up rate was 86%. In crude analysis regarding 90-day functional outcome, a favorable outcome was more often observed in patients receiving reversal treatment (*n* = 349; mRS 0–2: 15.4%, mRS 3: 10.3%, mRS 4: 14.9%, mRS 5: 12.2%, mRS 6: 33.6%) compared to those not receiving treatment (*n* = 174; mRS 0–2: 8.9%, mRS 3: 7.4%, mRS 4: 7.4%, mRS 5: 9.4%, mRS 6: 52.7%) (Figure 3). When analyzing crude functional outcome separately based on LOC category at admission (alert, drowsy, or comatose), a trend toward a more favorable outcome was seen in individuals receiving OAC-reversal treatment compared to no treatment (Supplemental Figures 1A–C). The proportion of patients with a favorable outcome was more distinct in patients with VKA-ICH who had received reversal treatment compared to no treatment, although this trend was not as clear regarding NOAC-ICH patients (Figure 4). Pre-stroke independent patients (*n* = 336) were more likely to remain independent at 90 days if reversal treatment was given, compared to no treatment (Supplemental Figure 2A). However, this trend was not as clear in pre-stroke dependent individuals in analysis of crude data (*n* = 210) (Supplemental Figure 2B).

The 90-day self-reported follow-up questionnaire was completed independently in 21.1% of cases, 39.6% required assistance, and 31.1% were completed by a caregiver alone. Patients lost to follow-up were alive and included 50 individuals receiving reversal treatment (13.6%) and 29 receiving no reversal treatment (14.3%).

## Discussion

In this large observational study on reversal treatment following OAC-ICH, we found that 35% of OAC-ICH patients did not receive reversal treatment. Our study shows improved survival and functional outcome in patients receiving reversal treatment compared to those who did not. Importantly, improved survival remained significant after adjusting for imbalances in relevant baseline data (*HR* = 0.68) and was thus not restricted to younger patients and several other predictors of good outcome in ICH.

Our findings regarding 90-day mortality are consistent with results from other studies (15, 27, 28). However, some studies have shown contradicting results, including a recent systematic review of 21 studies that did not show improved survival following the use of reversal agents in VKA-ICH (29). The sample size of each study included in this systematic review was small and confounded by limited data interpretation. Non-significant findings have also been observed in two multicenter observational studies concerning NOAC-ICH (10, 30). It is possible that these studies were underpowered or confounded by residual center effects.

Level of consciousness was strongly related to prognosis. This is not surprising, since LOC may be regarded as a proxy for stroke severity and hematoma volume (31). In our study, 90-day all-cause mortality in comatose patients was 82% in OAC-reversal patients and 95% in non-reversal patients, with low functional independence achieved in only 1.6–3% of cases. Comatose patients presented most often within 3 h of symptom onset, yet they represented only 9% of patients in the reversal treatment group. Early arrival within 3 h of symptom onset did thus not in itself determine treatment approach. In comparison, alert and drowsy patients received reversal treatment to a greater extent compared to comatose patients, suggesting a preference toward treating milder OAC-ICH, a practice that is not in line with recently published ESO guideline recommendations which promote treatment regardless of hemorrhagic stroke severity (18). Additional factors predictive of death included male sex, age, and intraventricular hemorrhage. Patients withheld reversal treatment were more often older, comatose, pre-stroke dependent, and taking NOAC. Clinical treatment decisions like withdrawal of care may have been based on individual physician perceptions on appropriateness of reversal therapy, thereby presuming that older and comatose patients did not receive reversal treatment to the same extent as patients with a seemingly better prognosis. Furthermore, the questionable efficacy of PCC in NOAC-ICH patients may have deterred physicians from using this intervention, thus accounting for the large number of NOAC-ICH patients who did not receive reversal treatment. Only few patients were treated with direct antidote Idarucizumab, and Andexanet alfa was not available during the inclusion period.

Patients receiving OAC reversal therapy had improved 90-day survival but had a greater proportion of dependency compared to non-reversed patients (mRS 3–5: 37.4 vs. 24.2%), which may be explained by the increased number of deaths in the non-reversed group (Figure 3). In a separate crude functional outcome analysis, functional outcome following reversal of NOAC did not show as clear of a trend toward a more favorable outcome as observed in patients who received VKA reversal. NOAC reversal may have been less effective due to the unavailability of specific antidotes for factor Xa inhibitors during the study period. Considering that the majority of patients in our study were on VKA treatment, reversal treatment may have been more effective in this group. Since imbalances in baseline data, specifically baseline mRS, were not adjusted for in 90-day functional outcome analyses and confounding by indication was unable to be accounted for, these results should be interpreted with caution.

### Strengths and Limitations

Riksstroke is a national database with >90% coverage, identifying the vast majority of OAC-related ICH cases in Sweden during 2017. The risk of selection bias is therefore estimated to be low. Secondly, the validity of this study is improved by the complete coverage of mortality status and few patients lost to 3-month follow-up (14%). Our data reflect recent clinical practice in use of reversal therapies in Sweden.

Several limitations of our study should be recognized. (A) The study was observational and retrospective. (B) There were imbalances in baseline data between the OAC reversal and non-reversal groups, suggesting the presence of confounding by indication (i.e., treatment decisions may have been based on individual physician perception of appropriateness of reversal therapy). Despite adjusting for all relevant confounders available in Riksstroke, the difference in survival may be influenced by residual confounding. The presumed efficacy of PCC for NOAC-ICH may be a result of residual confounding since previous studies have shown PCC to be ineffective in improving survival outcome in this patient group, hence the introduction of specific antidotes (10, 30). In line with this argument, our subgroup analysis of NOAC-ICH patients only showed a non-significant survival trend favoring patients who received reversal treatment compared to those who did not. We were unable to determine the effect on outcome related to the use of direct antidotes in NOAC-ICH patients since only few patients with Dabigatran related ICH had received Idarucizumab during our inclusion period. In addition, the direct antidote for factor Xa inhibitors, Andexanet alfa, was unavailable during the inclusion period. (C) The lack of radiologic data regarding hemorrhage volumes and hematoma expansion may account for discrepancies observed between our results and the results from aforementioned studies. However, LOC was exploited as a proxy for hemorrhage severity (31). (D) Data on clinical treatment decisions like withdrawal of care were not available. (E) Data on the dosage and timing of administration of the hemostatic agent were not available. (F) Data on acute blood pressure lowering were not available, an intervention that has shown to improve outcome (32). (G) Whereas 90-day functional outcome data are self-reported, a validation study has shown good agreement with objective data collection (25). However, only one third of patients could estimate their functional status independently, reflecting poor prognosis following OAC-ICH. Fourteen percent of individuals who did not answer the follow-up questionnaire were more often pre-stroke dependent and required lengthier hospital stays, suggesting that functional outcome rates may still be overestimated.

Hence, our results be should be interpreted with caution, and further studies are needed to more precisely determine the effects of reversal therapies in oral anticoagulant-related ICH.

## Conclusion

In this large national study reflecting recent (2017) clinical practice in treatment of OAC-related ICH in Sweden, 35% of OAC-ICH patients did not receive reversal therapy. Current ESO guidelines (2019) recommend the use of anticoagulant reversal treatment in OAC-ICH (18). Patients either on NOAC, or comatose at admission were less likely to receive OAC-reversal treatment. Overall, we found that patients receiving anticoagulant reversal treatment had superior 90-day survival compared to patients not receiving reversal treatment. Reversal treatment may be inappropriately withheld in a large proportion of patients. On the other hand, favorable prognosis (mRS 0–2) was observed in only 15% of OAC-ICH patients receiving reversal treatment, emphasizing the need for further studies on reversal therapies in specific groups of patients with stratification for known risk factors such as LOC, timing of treatment and ICH volume.

## Data Availability Statement

Requests to access the dataset supporting the conclusions of this article may be sent to Riksstroke after obtaining the appropriate ethics approval.

## Ethics Statement

The studies involving human participants were reviewed and approved by ethical approval for this study was obtained from the local Research Ethics Committee in Lund, Sweden (dnr 2017/529). Written informed consent for participation was not required for this study in accordance with the national legislation and the institutional requirements.

## Author Contributions

TA-H was involved in data analysis, researched literature, and wrote the first draft of the manuscript. TA-H and TU were involved in gaining ethical approval. TA-H, TU, MP, BN, and JP reviewed and edited the manuscript and approved the definitive version of the manuscript. TA-H, TU, BN, and JP conceived the study. All authors contributed to the article and approved the submitted version.

## Conflict of Interest

The authors declare that the research was conducted in the absence of any commercial or financial relationships that could be construed as a potential conflict of interest.
