# Supplementary material for: Reversal Treatment in Oral Anticoagulant-Related Intracerebral Hemorrhage—An Observational Study Based on the Swedish Stroke Register
Source: Front Neurol. 2020 Jul 29;11:760. doi: 10.3389/fneur.2020.00760 (PMC7438936; doi:10.3389/fneur.2020.00760)
Supplement: Supplementary file 1 [file Data_Sheet_1.docx]

| Supplemental Table 1. Baseline characteristics comparing 341 patients followed up and lost to follow up at 90 days post-ICH. Proportion of missing data varied between 0 and 1.0% for all variables, except for VKA reversal type (2.3%) intraventricular hemorrhage (2.5%), pre-stroke dependency (4.5%), NOAC reversal type (11%), and time interval between symptom onset to hospital arrival (8.8%). | | | |
| --- | --- | --- | --- |
| Variables | Lost to FU | Followed up |  |
|  | (n=79) | (n=262) |  |
|  | n (%) | n (%) | p-value |
| **Demographics** | | | |
| Mean age | 79.2 (11.6)* | 77.8 (8.9)* | 0.27 |
| Sex (male) | 36 (45.6) | 141 (53.8) | 0.20 |
| Pre-stroke dependent | 38 (48.1) | 79 (30.3) | 0.003 |
| **Vascular risk factors** | | | |
| Hypertension | 64 (81.0) | 220 (84.0) | 0.68 |
| Atrial fibrillation | 69 (87.3) | 227 (86.6) | 0.86 |
| Diabetes | 19 (24.1) | 51 (19.6) | 0.39 |
| Previous stroke | 23 (29.1) | 80 (30.5) | 0.81 |
| Previous TIA | 11 (13.9) | 31 (11.9) | 0.82 |
| **Clinical characteristics** |  |  |  |
| Time interval between symptom onset to hospital arrival |  |  | 0.09 |
| *0 – 3 h* | 24 (30.4) | 98 (37.4) |  |
| *3 – 6 h* | 26 (32.9) | 66 (25.2) |  |
| *> 6 h* | 18 (22.8) | 79 (30.2) |  |
| Admitted to stroke unit or ICU | 70 (88.6) | 242 (92.4) | 0.29 |
| Median days of hospital admittance | 13 | 12 | 0.53 |
| Level of consciousness at hospital admission |  |  |  |
| *Alert* | 62 (78.5) | 205 (79.2) | 0.90 |
| *Drowsy* | 17 (21.5) | 45 (17.4) | 0.41 |
| *Comatose* | 0 (0.0) | 9 (3.5) | 0.09 |
| **Hemorrhage location** |  |  |  |
| Supratentorial | 70 (88.6) | 226 (86.3) | 0.59 |
| *Intraventricular hemorrhage* | *26/70 (37.1)* | *73/226 (33.2)* | 0.54 |
| *Neurosurgery* | *0/70 (0.0)* | *7/226 (3.1)* | 0.14 |
| Infratentorial | 9 (11.4) | 33 (12.6) | 0.78 |
| *Intraventricular hemorrhage* | *2/9 (22.2)* | *5/33 (15.6)* | 0.64 |
| *Neurosurgery* | *2/9 (22.2)* | *3/33 (9.1)* | 0.28 |
| **Anticoagulant** |  |  |  |
| NOAC | 26 | 111 |  |
| *Apixaban* | 19 (73.1) | 79 (71.2) | 0.82 |
| *Rivaroxaban* | 6 (23.1) | 24 (21.6) |  |
| *Dabigatran* | 1 (3.8) | 8 (7.2) |  |
| VKA | 53 | 151 |  |
| *INR < 1.7* | 6 (11.3) | 14 (9.3) | 0.28 |
| *INR 1.7 – 3* | 26 (49.1) | 93 (61.6) |  |
| *INR > 3* | 21 (39.6) | 44 (29.1) |  |
| *Standard deviation of the mean. Abbreviations: OAC = oral anticoagulant, ICH = intracerebral haemorrhage, VKA = Vitamin-K Antagonist, NOAC = Non-Vitamin K Oral Anticoagulant, TIA = transitory ischemic attack, INR = international normalized ratio, ICU = intensive care unit. | | | |

| Supplemental Table 2. Cox regression analysis stratified for level of consciousness showing Hazard Ratios (HR) for 90-day mortality in 572 patients with OAC-ICH. Including NOAC vs VKA. Simple analysis is displayed as a crude model. | | | | |
| --- | --- | --- | --- | --- |
| Variable | HR | 95% CI | | P-value |
|  |  | lower | upper |  |
| **Crude model (non-stratified)** |  |  |  |  |
| No OAC reversal | 1.92 | 1.48 | 2.49 | <0.001 |
| **Adjusted model (stratified)*** |  |  |  |  |
| No OAC reversal | 1.49 | 1.08 | 2.06 | 0.01 |
| NOAC | 0.95 | 0.70 | 1.28 | 0.73 |
| Male sex | 1.42 | 1.06 | 1.91 | 0.02 |
| Age | 1.05 | 1.02 | 1.07 | <0.001 |
| Diabetes | 1.04 | 0.71 | 1.52 | 0.86 |
| Hypertension | 0.80 | 0.57 | 1.14 | 0.22 |
| Atrial fibrillation | 0.73 | 0.47 | 1.14 | 0.16 |
| Pre-stroke dependency | 1.02 | 0.74 | 1.41 | 0.91 |
| Intraventricular hemorrhage | 2.41 | 1.77 | 3.29 | <0.001 |
| Neurosurgery not performed | 2.15 | 0.91 | 5.06 | 0.08 |
| Infratentorial hemorrhage | 1.46 | 0.96 | 2.23 | 0.08 |
| Abbreviations: OAC = oral anticoagulant, ICH = intracerebral hemorrhage, CI = confidence interval.  *Stratified for level of consciousness. | | | | |
